# Qualitative and Quantitative Analysis of the Product and By-Products from Transesterification between Phenol and Dimethyl Carbonate

**DOI:** 10.1155/2019/7632520

**Published:** 2019-03-06

**Authors:** Tao Liu, Jing Hu, Li Yong, Gang Zhang, Yi Zhang, Tong Chen, Gongying Wang

**Affiliations:** ^1^Chengdu Institute of Organic Chemistry, Chinese Academy of Sciences, Chengdu 610041, China; ^2^National Engineering Laboratory for VOCs Pollution Control Material & Technology, University of Chinese Academy of Sciences, Beijing 101408, China; ^3^Sichuan Center for Disease Control and Prevention, Chengdu 610041, China

## Abstract

By-products (phenyl salicylate, phenyl 4-hydroxybenzoate, and xanthone) from transesterification between phenol and dimethyl carbonate (DMC) were qualitatively analyzed by gas chromatography-mass spectrometry, and a gas chromatographic method with directed injection for simultaneous quantitative analysis of the product (DPC) and by-products of the transesterification has been established. Based on the results of qualitative and quantitative analyses, the mechanism of the by-products generation was preliminarily deduced. The sample for quantitative analysis was directly diluted in acetone, and related compounds were separated on an HP-5 capillary column and detected by a hydrogen flame ionization detector (FID). The product and by-products were well separated, the correlation coefficients (*r*) within the concentration range of 1.0 *μ*g/mL–100 *μ*g/mL were ≥0.9997, the relative standard deviations were between 0.5% and 4.4%, spiked recoveries were between 91.5% and 105.6%, and detection limits were between 0.11 and 0.18 *μ*g/mL. The established method is simple, rapid, accurate, sensitive, and highly specific. It is suitable for simultaneous qualitative and quantitative analyses of the product and by-products of transesterification between phenol and DMC.

## 1. Introduction

Nontoxic and environmentally friendly diphenyl carbonate (DPC) is an important “green” chemical product, which may be used for synthesizing many important organic compounds and polymer materials [1, 2] and particularly, can, superseding highly toxic phosgene, react with bisphenol A to produce polycarbonate (PC) with excellent performance [3, 4]. Since PC is extensively used in mechanical manufacture, electronic appliances, military and lighting industries, security, medical devices, etc., the demand for the raw material for PC synthesis, DPC, has been rising sharply and, as a result, synthetic processes of DPC have become a hot research field in recent years [5, 6]. DPC is conventionally synthesized by the phosgene method. However, phosgene is highly toxic. HCl, a by-product of the synthesis method, can corrode the equipment and pollute the environment. Additionally, chlorine-containing impurities will be present in the product [7, 8]. For these reasons, the phosgene method has been gradually replaced by other methods. Phosgene-free approaches for synthesis of DPC mainly include oxidative carbonylation of phenol and transesterification. In oxidative carbonylation of phenol, DPC is synthesized from phenol, O_2_, and CO by a one-step reaction under the catalytic effect of Pd. The used raw materials are cheap and easily available. The process is simple, and the atom utilization rate is high. However, the used Pd catalyst is relatively complicated and expensive. Its activity and selectivity are relatively low, and its useful life is short [9, 10]. The method involving transesterification between phenol and DMC has the advantages of a simple process, high atom utilization rate, and high selectivity. Additionally, the catalyst is cheap and easily available. Therefore, it is considered an industrialized synthetic route with the most promising application prospect for production of DPC at present [11–14].

During transesterification between phenol and DMC, the composition of product and by-products varies depending on the synthetic process and catalyst [15, 16]. Some rearrangement products with high boiling points approximate that of DPC, which cannot be easily isolated in later purification steps, are prone to remain in DPC. They will reduce the DPC purity and exert significant influence on the quality of PC synthesized in the next step [17–19]. To obtain high-purity DPC and high-quality PC, the content of each by-product impurity in DPC must be controlled at levels below 100 *μ*g/g [20]. Methods for quantifying the reaction product have been reported [21, 22]. However, no report on qualitative and quantitative analysis of the trace by-products is available. In addition, gas chromatography is the best method for the determination of volatile or semivolatile components [23]. For the purpose of in-depth exploration of the generation mechanism, influencing factors, distribution, and control of the rearrangement products is generated in the reaction. Based on previous studies of the transesterification [24–29], a method has been established for qualitative and quantitative analysis of the product and rearrangement products of transesterification between phenol and DMC.

## 2. Experimental

### 2.1. Instruments and Reagents

The following instruments were used in this study: a 7820A gas chromatograph (Agilent Technologies, USA) equipped with a HP-5 column (30 m × 0.32 mm × 0.25 *μ*m, 5% phenyl methyl siloxane) (Agilent Technologies, USA); a HP 6890/5973 gas chromatograph-mass spectrometer (Agilent Technologies, USA) equipped with a HP-5 MS column (30 m × 0.32 mm × 0.25 *μ*m); a DF-101S heat-collection constant-temperature magnetic stirrer (Gongyi Yuhua Instruments Co., Ltd, China); and a GS-0.1 high-pressure batch reactor (Weihai Chemical Machinery Co., Ltd, China). Phenyl salicylate (GC) was purchased from Alfa Aesar (USA); xanthone (GC) from J&K Scientific Ltd. (China); phenyl 4-hydroxybenzoate (GC) from TCI (Shanghai) Development Co., Ltd (China); DPC (GC) from Nanjing Duly Biotech Co., Ltd (China); DMC (≥99%) and phenol (GC) from Aladdin (USA); anisole (GC) from Chengdu Kelong Chemical Reagents Plant (China); acetone (HPLC) from Scientific (USA); dibutyltin oxide (AR) from Acros (Belgium); butyltin hydroxide (AR) and tributyltin chloride (AR) from Merck (Germany); and bis(tributyl)tin oxide (AR), dibutyltin dilaurate (AR), and butyltin trichloride (AR) from Alfa Aesar (USA). Methyl phenyl carbonate (MPC, ≥99%) was prepared by our laboratory.

### 2.2. Transesterification Approach for Synthesis of DPC

Normal-pressure synthesis of DPC: transesterification was carried out by a reported method [27] with modifications: a 100 mL three-necked flask equipped with a thermometer and a fractionating column filled with small porcelain rings was fixed on a heat-collection constant-temperature magnetic stirrer. Under normal pressure, a quantity of phenol, dibutyltin oxide (catalyst), and DMC was added, and nitrogen was charged to expel air in the reactor. Then, the temperature was increased with stirring. During the beginning stage of the reaction, the operation was performed under a fully refluxing condition. When the temperature at the top of the tower reached the azeotropic point of DMC and methanol, collection of the distillate started. Pressurized synthesis of DPC: under normal pressure, a quantity of phenol, dibutyltin oxide (catalyst), and DMC was added into a GS-0.1 high-pressure batch reactor, which was then fixed in a temperature-controllable mantle. Nitrogen was charged for pressurization, and the temperature was increased with stirring to synthesize DPC.

### 2.3. Analytical Methods

The samples were quantitatively analyzed on a 7820A gas chromatograph under the following analytical conditions. High-purity nitrogen was used as the carrier gas. The column flow rate was 2.5 mL/min. The makeup nitrogen flow rate was 25 mL/min. The air flow rate was 400 mL/min. The hydrogen flow rate was 30 mL/min. Samples were injected in a split mode with a split ratio of 50 : 1. The injection volume was 0.4 *μ*L. The injection port temperature was 280°C. The detector temperature was 300°C. Programmed temperature increase was performed: the initial temperature was 120°C, and the temperature was increased at a rate of 20°C/min to 280°C and held at 280°C for 5 min. Samples were qualitatively analyzed on a HP 6890/5973 gas chromatograph-mass spectrometer. Analytical conditions of gas chromatography: the injection port temperature was 260°C; the column temperature was held at 100°C for 2 min, increased at a rate of 10°C/min to 280°C, and held at 280°C for 5 min; the column flow rate was 0.8 mL/min; samples were injected in a split mode with a split ratio of 50 : 1; and the injection volume was 0.2 *μ*L. Analytical conditions of mass spectrometry: the ionization source was an EI source; the electron energy was 70 eV; the ion source temperature was 230°C; the transmission line temperature was 280°C; the quadrupole temperature was 150°C; the degree of vacuum was lower than 1.33 × 10^−5^ Pa; the mass scanning range was 20–450 amu (M/Z); the resolution was 5000; and the solvent delay was 2.10 min. All samples were directly injected following dilution in acetone and quantitatively analyzed by the external standard method.

### 2.4. Quality Control

Samples were preserved in tightly closed containers and stored at low temperatures with light protection. Determination was carried out immediately after synthesis of DPC from transesterification. Prior to each determination, reagent blank and laboratory blank were analyzed. The quality control sample was injected following injection of every 10 samples, and the deviation was within ±10% of the standard value.

## 3. Results and Discussion

### 3.1. Qualitative Analysis of the Product and By-Products from Transesterification

**Figure 1:**
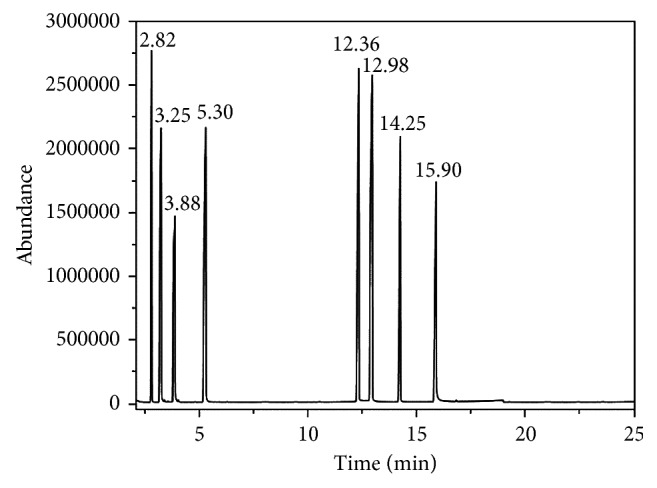
The total ion current chromatogram of product and by-products from the transesterification between phenol and DMC.

The product and by-products of transesterification between phenol and DMC catalyzed by dibutyltin oxide were separated on a gas chromatograph-mass spectrometer. The total ion chromatogram is shown in Figure 1. It can be known from Figure 1 that the substances were well separated and no mutual interference was present. Characteristic fragment ion peaks and matching degrees of all compounds were comparatively analyzed by library searching. Compounds at retention times of 2.82 min and 3.25 min were identified as reactant DMC and anisole, respectively. Compounds at retention times of 3.88 min, 12.98 min, 14.25 min, and 15.90 min were identified as reactant phenol and by-products phenyl salicylate, xanthone, and phenyl 4-hydroxybenzoate, respectively. The compound at a retention time of 5.30 min was identified as intermediate MPC. The compound at a relative retention time of 12.36 min was identified as the primary product DPC. A mixed standard solution containing the above eight compounds in acetone was analyzed on the gas chromatograph-mass spectrometer. The analytical results corresponded to the above qualitative analysis results.

### 3.2. Mechanisms of Generation of the Product and By-Products from Transesterification

**Figure 2:**
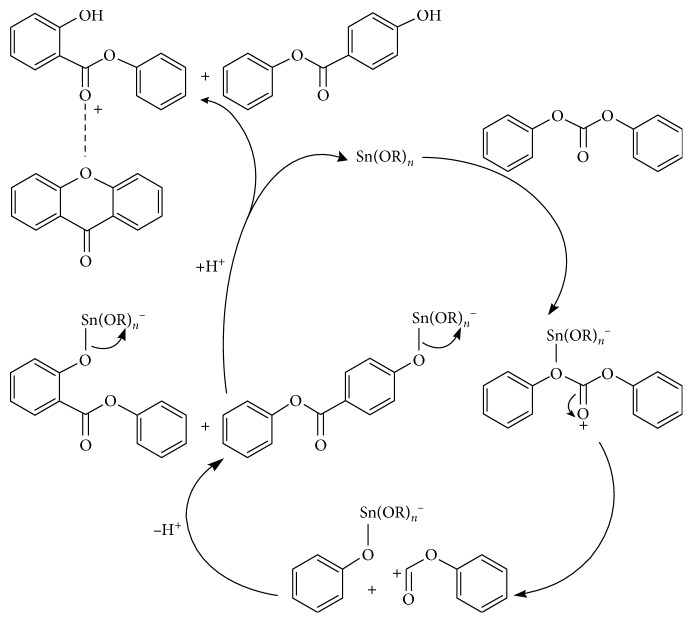
The mechanism of by-products generation from the transesterification between phenol and DMC.

According to the previous studies [25, 30], the transesterification between phenol and DMC comprises the following two steps: in the first step, DMC is transesterified with phenol into intermediate MPC, as shown in reaction equation (1.1); in the second step, MPC is transesterified with phenol into the target product DPC, as shown in reaction equation (1.2), or MPC is self-disproportioned into DPC and DMC, as shown in reaction equation (1.3). A parallel reaction, i.e., methylation of phenol with DMC into by-product anisole, will occur during the transesterification, as shown in reaction equation (1.4). Other by-products including phenyl salicylate, xanthone, and phenyl 4-hydroxybenzoate were also found in the qualitative analysis. Taking into account the similar molecular structures between by-products and DPC [31, 32] and the Fries rearrangement reaction mechanism [33–35], the mechanism of generation of the three by-products was preliminarily deduced. DPC has a phenolic ester structure. Therefore, similar to the C-O Fries rearrangement reaction, a rearrangement reaction consisting of two steps will occur while DPC is heated under the effects of Lewis acid catalysts (such as dibutyltin oxide, butyltin hydroxide, and so on), as shown in reaction equations (1.5) and (1.6), and generates by-products phenyl salicylate, xanthone, and phenyl 4-hydroxybenzoate. The mechanism of generation is shown in Figure 2: in the first step, carboxyl oxygen atoms from DPC and tin atoms with Lewis acidity are coordinated to form organotin group. Then, the organotin group is rearranged on phenol oxygen atoms, and C-O single bond is cleaved, generating phenolic group stannide and acyl carbocations. Subsequently, electrophilic substitution occurs between acyl carbocations and *o*- or *p*-positions of phenolic groups on benzene rings and then hydrolyzed to form phenol salicylate and phenyl 4-hydroxybenzoate. The proportions of generated *o*- and *p*-position products depend on the molecular structure, reaction conditions, and catalysts [36]. Among them, temperature is the main factor. Low temperatures will lead to *p*-position product phenyl 4-hydroxybenzoate and high temperatures to *o*-position product phenol salicylate. In the second step, phenol salicylate is further rearranged into 2,2′-dihydroxyl diphenylketone and, following dehydration and ring-closure reaction, xanthone is generated:

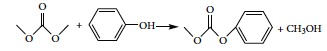
(1.1)

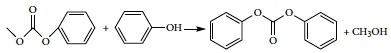
(1.2)

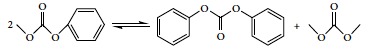
(1.3)

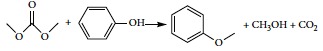
(1.4)

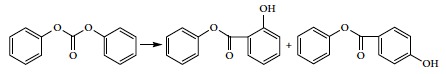
(1.5)

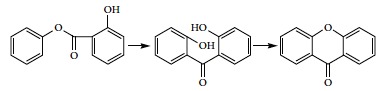
(1.6)


### 3.3. Methodological Validation for Quantitative Analysis of the Product and By-Products from Transesterification

#### 3.3.1. Linearity, Detection Limits, and Limits of Quantitation

**Figure 3:**
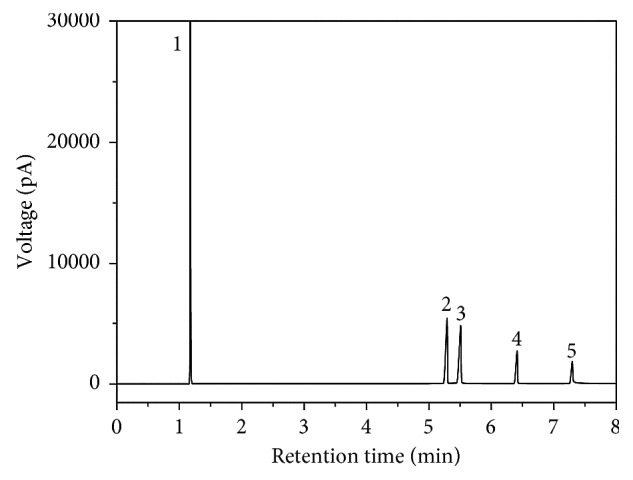
Chromatogram of mixed standard solution with gas chromatography. 1, acetone; 2, DPC; 3, phenol salicylate; 4, xanthone; 5, phenyl-4-hydroxybenzoate.

Serial mixed standard solutions containing 1.0, 2.5, 5.0, 10.0, 50.0, 100, and 500 *μ*g/mL of DPC, phenol salicylate, phenyl 4-hydroxybenzoate, and xanthone in acetone were prepared and successively injected. The chromatogram of a standard solution is shown in Figure 3. It can be known that the product and by-products were well separated. Ten copies of the mixed standard solution approximate to the reagent blank were injected. The detection limits of standard solutions were defined as three times the standard deviations and limits of quantitation of standard solutions as four times the detection limits of standard solutions. Detection limits and limits of quantitation of the method were calculated based on a sampled volume of 1.00 mL and a finally diluted volume of 10.00 mL. It can be known from Table 1 that, for the product DPC and by-products phenol salicylate, phenyl 4-hydroxybenzoate, and xanthone, good linearity was demonstrated over the concentration range of 1.0 to 500 *μ*g/mL. The detection limits of the method were between 0.11 and 0.18 *μ*g/mL. The limits of quantitation were between 0.44 and 0.72 *μ*g/mL. The method has a broad linear range and is highly sensitive. It is a suitable simultaneous determination of the product and three trace by-products of the transesterification.

#### 3.3.2. Specificity

**Figure 4:**
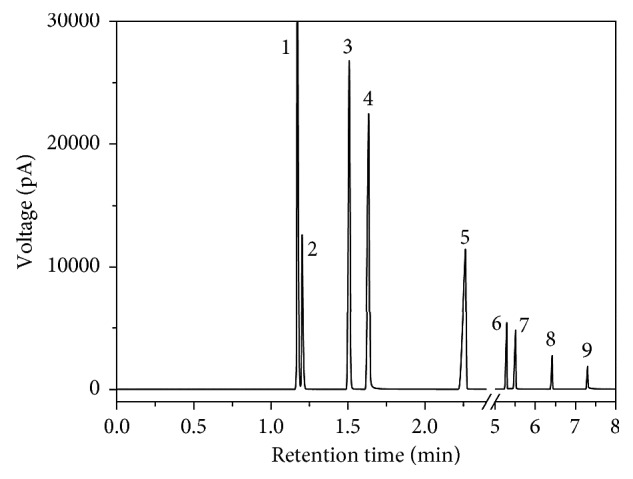
Chromatogram of mixed standard solutions and four kinds of distracters. 1, acetone; 2, DMC; 3, anisole; 4, phenol; 5, MPC; 6, DPC; 7, phenol salicylate; 8, xanthone; 9, phenyl 4-hydroxybenzoate.

Four interferents including reactant DMC and phenol, intermediate MPC, and by-product anisole of five times its concentration of 10.0 *μ*g/mL mixed standard solution were added, and then the mixed standard solution was injected. The obtained chromatogram shows in Figure 4 that the peaks of analytes and interferents were completely separated and no mutual interference was present. To investigate the matrix interference, actual transesterification samples and actual spiked samples were analyzed by this method. It was found that the chromatographic peaks were well separated and the matrix had not exhibited interference, indicating the established analytical method is well resistant to interference.

#### 3.3.3. Precision and Accuracy

Mixed standard solutions of low, middle, and high concentrations within the standard linear range were separately added to eleven sample solutions approximate to the reagent blank and analyzed by the method described in Section 2.3. Analytical results were calculated according to the standard curve equations, and the relative standard deviations were calculated to assess the method precision. Mixed standard solutions of low, middle, and high concentrations were added to sample solutions of known analyte concentrations and analyzed by the same method. The measured increments were compared to the actual additions to assess the method accuracy. The results are presented in Table 2 that the spiked recoveries of this method were between 91.5% and 105.6% and the relative standard deviations were between 0.5% and 4.4%.

#### 3.3.4. Practical Application

**Figure 5:**
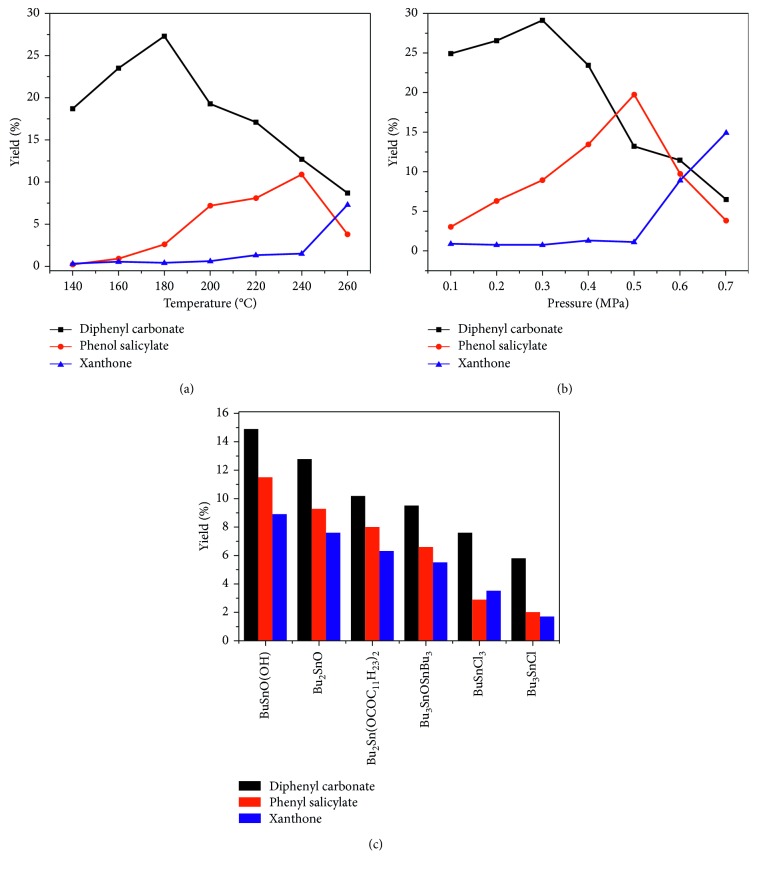
The effects of temperature (a), pressure (b), and ligands of organic tin catalyst (c) on the product and by-products from transesterification. Reaction conditions: (a) phenol: 10 g; *n*(phenol) = *n*(DMC); catalyst (dibutyltin oxide): 0.20 g; reaction time: 12 h; and reaction pressure: 0.3 MPa. (b) Phenol: 10 g; *n*(phenol) = *n*(DMC); catalyst (dibutyltin oxide): 0.20 g; reaction time: 12 h; and reaction temperature: 180°C. (c) Phenol: 10 g; *n*(phenol) = *n*(DMC); catalyst: 0.20 g; reaction time: 12 h; reaction temperature: 240°C; and reaction pressure: 0.5 MPa.

Products and by-products from the transesterification at different reaction temperatures and under different reaction pressures were quantitatively analyzed by the method. Yields of DPC and the three by-products were calculated. The obtained results are presented in Figure 5. It can be known from Figure 5(a) that, in the initial stage, the yield of DPC rose gradually with increase in the reaction temperature. When the temperature reached 180°C, the yield began to decrease. When the temperature was below 160°C, no phenol salicylate was generated. The yield of phenol salicylate rose with the increase in the temperature between 160°C and 240°C. When the temperature increased to 240°C, the yield began to decrease. The yield of xanthone was very low at temperatures below 240°C but began to rise when the temperature increased more than 240°C. In addition, no phenyl 4-hydroxybenzoate was generated at temperatures above 140°C. It is thus deduced that, during the initial stage of the transesterification reaction, DPC is first generated. With the temperature increases, the reaction proceeds in the forward direction as DPC rearrangement is an endothermic reaction, gradually generating by-product phenol salicylate. With the continued increase of the reaction temperature, the reaction continues in the forward direction, and the generated phenol salicylate is rearranged and dehydrated, generating xanthone. It can be known from Figure 5(b) that, the yield of DPC gradually increased in the initial stage. With gradual increase of the reaction pressure, the reaction proceeds in the direction of generating phenol salicylate, which has a smaller spatial structure than DPC, so that the yield of DPC begins to decrease and the yield of phenol salicylate gradually increases. With the continued increase of the reaction pressure, the reaction proceeds in the direction of generating xanthone because of an even smaller spatial structure than phenol salicylate, so that the yield of DPC further decreases, the yield of phenol salicylate also begins to decrease, and the yield of xanthone gradually increases. The impacts of reaction temperature and pressure on generation of phenol salicylate, xanthone, and phenyl 4-hydroxybenzoate have also further verified the mechanisms of generation of the by-products described in Section 3.2. The impacts of the ligands of organic tin on generation of by-products were also investigated. It can be known from Figure 5(c) that the yields of DPC, phenol salicylate, and xanthone are in the following descending order: BuSnO(OH) > Bu_2_SnO > Bu_2_Sn(OCOC_11_H_23_)_2_ > Bu_3_SnOSnBu_3_ > BuSnCl_3_ > Bu_3_SnCl. This is mainly because organic tin catalysts used in the transesterification reaction are Lewis acids and tin atoms are the catalyst active center. The smaller the steric hindrance of the groups coordinated with tin atoms is, the higher the reactivity of the catalyst is, and the more favorable it is for generation of by-products including phenol salicylate and xanthone. Moreover, the stronger the electrophilic effect of the coordinating groups is, the stronger the acidity of tin atoms is, the higher the activity of the catalyst is [26], and the more favorable it is for generation of by-products. Additionally, the order of yields of by-products corresponding to bis(tributyl)tin oxide and butyltin trichloride also indicates the electrophilic effect of coordinating groups is stronger than the steric hindrance.

## 4. Conclusion

In this study, the product and by-products from transesterification between DMC and phenol were qualitatively analyzed on a gas chromatograph-mass spectrometer. Based on the analytical results, the mechanism of generation of the by-products was preliminarily deduced, and a gas chromatographic method with directed injection for simultaneous quantitative analysis of the reaction product and by-products was established. The established method is simple, rapid, accurate, sensitive, and highly specific. It is suitable for simultaneous qualitative and quantitative analyses of the product and by-products of transesterification between phenol and DMC. It can provide vigorous data support for controlling of the by-products in pilot and industrialized transesterification between phenol and DMC and purity analysis of the product DPC.

## Figures and Tables

**Table 1:** Linear equations, linear correlation coefficients, detection limits, and limits of quantitation.

Compound	Linear equations	Correlation coefficients	Detection limits (*μ*g/mL)	Limits of quantitation (*μ*g/mL)
DPC	*y* = 2341.7*x* + 171.88	0.9998	0.15	0.60
Phenol salicylate	*y* = 2624.8*x* − 803.01	0.9997	0.11	0.44
Phenyl 4-hydroxybenzoate	*y* = 2500.5*x* + 922.01	0.9999	0.12	0.48
Xanthone	*y* = 2101.3*x* − 133.25	0.9998	0.18	0.72

**Table 2:** Recoveries and relative standard deviations of the product and by-products from transesterification.

Compound	Recoveries (%)			Relative standard deviations (%)		
	1.0 *μ*g/mL	20.0 *μ*g/mL	400 *μ*g/mL	0.50 *μ*g/mL	10.0 *μ*g/mL	500 *μ*g/mL
DPC	93.1	102.2	95.4	4.2	1.7	0.8
Phenol salicylate	99.9	100.2	105.6	2.4	3.9	0.5
Phenyl 4-hydroxybenzoate	96.7	98.6	104.1	3.0	0.9	2.3
Xanthone	91.5	94.8	95.0	4.4	2.2	1.0

## Data Availability

The data used to support the findings of this study are included within the article.
